# Associations of neighbourhood walkability indices with weight gain

**DOI:** 10.1186/s12966-018-0668-2

**Published:** 2018-04-03

**Authors:** Mohammad Javad Koohsari, Koichiro Oka, Ai Shibata, Yung Liao, Tomoya Hanibuchi, Neville Owen, Takemi Sugiyama

**Affiliations:** 1 Faculty of Sport Sciences, Waseda University, 2-579-15 Mikajima, Tokorozawa, Saitama, 359-1192 Japan; 2 Behavioural Epidemiology Laboratory, Baker Heart and Diabetes Institute, Melbourne, Australia; 3 Mary MacKillop Institute for Health Research, Australian Catholic University, Melbourne, Australia; 4 Faculty of Health and Sport Sciences, University of Tsukuba, Tsukuba, Japan; 5 Department of Health Promotion and Health Education, National Taiwan Normal University, Taipei City, Taiwan; 6 School of International Liberal Studies, Chukyo University, Nagoya, Japan; 7 Swinburne University of Technology, Melbourne, Australia

**Keywords:** Built environment, Obesity, Urban design, Public health, Space syntax

## Abstract

**Background:**

Inconsistent associations of neighbourhood walkability with adults’ body weight have been reported. Most studies examining the relationships of walkability and adiposity are cross-sectional in design. We examined the longitudinal relationships of two walkability indices – conventional walkability and space syntax walkability, and their individual components, with weight change among adults over four years.

**Methods:**

Data were from the Physical Activity in Localities and Community study in Adelaide, Australia. In 2003–2004, 2650 adults living in 154 Census Collection Districts (CCDs) returned baseline questionnaires; in 2007–2008, the follow-up survey was completed by 1098. Participants reported their weight at baseline and at follow-up. Neighbourhood walkability indices were calculated using geographic information systems and space syntax software. Linear marginal models using generalized estimating equations with robust standard errors were fitted to examine associations of the two walkability indices and their individual components with the weight at follow-up, adjusting for baseline weight, socio-demographic variables, and spatial clustering at the level of CCD.

**Results:**

The overall mean weight gain over four years was 1.5 kg. The two walkability indices were closely correlated (*r* = 0.76, *p* < 0.01). No significant associations were found between the overall neighbourhood walkability indices and weight change. Among walkability components, there was a marginally significant negative association between space syntax measure of street integration and weight change: one standard deviation increment in street integration was associated with 0.31 kg less weight gain (*p* = 0.09).

**Conclusions:**

Using a prospective study design and a novel space-syntax based measure of walkability, we were not able to identify relationships between neighbourhood walkability with weight gain. This is consistent with other inconclusive findings on the built environment and obesity. Research on the built environment and adults’ weight gain may need to consider not just local environments but also a larger scale environment within a city or workplace environment in order to capture multiple behaviours relevant to weight gain.

## Introduction

Despite the efforts to modify individual level factors that influence physical activity and diets, little progress has been made in reducing obesity. For example, the rate of obesity has been more than doubled since 1980 [1]. A multi-level approach that encompasses individual, social, environmental, and policy dimensions is needed to effectively address obesity [2]. There is an increasing interest in exploring the role of the built environment on obesity over the past decade [3, 4]. The built environment would be postulated to influence obesity at the population level by providing residents with opportunities to be more physically active [5, 6].

It is possible to hypothesize that neighbourhood walkability is related to weight gain as studies have consistently shown associations of this construct with physical activity. The walkability index consisting of four components (net residential density, intersection density, land use mix, and net retail area ratio) has been found associated with physical activity in countries such as the USA [7], Australia [8], Canada [9], and Belgium [10]. However, a recent review showed that less than half of the studies examining the relationships between walkability and weight status found significant associations [4]. In addition, most studies examining the relationships of walkability and obesity have been cross-sectional in design [11, 12]. In order to better understand how neighbourhood walkability influences residents’ weight status, evidence from longitudinal studies is needed. Furthermore, it is of interest to examine how a newly proposed measure of walkability, space syntax walkability (SSW), is associated prospectively with weight change. The advantages of SSW compared with the conventional 4-component measure of walkability have been explained in details elsewhere [13]. Since SSW employs a space syntax measure of integration, which is conceptually different from intersection density, SSW may be differentially associated with weight gain than is the conventional 4-component measure of walkability.

Using a prospective observational design, we examined how conventional walkability and SSW and their individual components were each associated with weight change over four years among Australian adults.

## Methods

### Data source and participants

Data were from the PLACE (Physical Activity in Localities and Community Environments) study conducted in Adelaide, Australia. The original aim of the PLACE study was to examine associations of neighbourhood environmental attributes with physical activity. Detailed methods of study design and sampling procedures have been documented elsewhere [8]. Briefly, residential addresses were randomly selected from 154 Census Collection Districts (CCD, a geographical unit comprising of about 250 households) within the city of Adelaide based on walkability and area-level socioeconomic status. The median size of the CCDs was 22 ha (interquartile range: 16 ha). In 2003–2004, a total of 2650 adults aged between 20 and 66 years old completed and returned the baseline questionnaire. The response rate was 11.5% (as a proportion of the households initially identified). The low response rate was partly because of having households rather than individuals as the sampling units. According to Census data [14], over 25% of selected households might have been ineligible based on the age criterion (20–65 years). But, due to the nature of the sampling structure, such potentially-ineligible households were not removed from the mailing list. Therefore, the actual response rate might have been around 35%, which is within the appropriate response rate ranges for mailing surveys in public health research [15]. The return rate for those who completed the postal survey, as a proportion of those who were known to be contacted, was 74.2%. Of these, 1098 completed the follow-up survey four years later (41.4% of the baseline participants). The Behavioural and Social Sciences Ethics Committee of the University of Queensland approved the study.

### Measures

#### Outcome variable

The outcome variable was weight change over four years calculated using self-reported weight at follow-up adjusting for baseline weight, which is equivalent to modelling weight change and controls for regression to the mean [16, 17].

#### Neighbourhood walkability

This study used two walkability indices: conventional 4-component walkability and SSW. Conventional walkability was calculated using geographic information systems (GIS) for each CCD, as a composite measure consisting of net residential density, intersection density, land use mix, and net retail area ratio [18]. Net residential density was calculated as the ratio of the number of dwelling units to the land area for residential use within each CCD. Intersection density was defined as the ratio of the number of intersections to the area of a CCD. Land use mix was as an entropy index describing the heterogeneity of five land uses (residential, commercial, recreational, industrial, and other) within a CCD [19]. Net retail area ratio was calculated as the ratio of the retail floor space to the retail parcel area. All scores were standardized. SSW was calculated as a composite measure of gross population density and street integration [13]. Gross population density was the ratio of the number of residents to the land area of each CCD. Street integration was calculated using street centreline data and Axwoman [20] and DepthMap [21]. Street integration refers to how a street is connected to other streets within the network. First, an integration score was calculated for each street segment considering all the other streets within a 1 km distance from its centre. Then, for each CCD, the mean street integration score was calculated for all street segments within the CCD. All scores were standardized.

#### Socio-demographic variables

Participants reported their age, gender, educational attainment, work status, marital status, having children in the household, annual household income, and car ownership. Socioeconomic status (SES) of each CCD was also identified using its median household weekly income, and all CCDs were dichotomized into a lower or higher SES category using the median.

#### Statistical analysis

Linear marginal models using generalized estimating equations with robust standard errors were fitted to examine associations of two walkability indices and their individual components with the weight at follow-up, adjusting for baseline weight, socio-demographic variables, and the spatial clustering at the level of CCD. Stata 14.0 (Stata Corp, College Station, Texas) was used to conduct the analyses.

## Results

The final study sample included 910 adults, after excluding those with missing data. Consistent with previous studies [22, 23], we also removed those with extreme weight change larger than 20 kg increase or decrease (over 5 kg/year). The baseline weight for those who participated in the follow-up, and those who dropped from the follow-up were 74.9 kg and 75.0 kg, respectively.

Table 1 shows the characteristics of the sample at baseline. The overall mean weight gain over 4 years was 1.5 kg, which is consistent with the reported weight increase in Australia [24]. The correlation between conventional walkability and SSW was 0.76 (*p* < 0.01).

**Table 1:** Sample characteristics at baseline (*N* = 910)

Variable	Mean (SD) or N (%)
Age (years)	48.7 (10.3)
Gender	
Women	556 (61.1%)
Education	
Tertiary or higher	424 (46.6%)
Children in household	
Yes	266 (29.2%)
Marital status	
Single	326 (35.8%)
Couple	567 (62.3%)
Other	17 (1.9%)
Household income (AUD$ per annum)	
< $20,800	167 (18.4%)
$20,800–41,599	222 (24.4%)
$41,600–77,999	310 (34.1%)
≥ $78,000	183 (20.1%)
Missing	28 (3.1%)
Car ownership	
No car	57 (6.3%)
One car	350 (38.5%)
Two or more cars	503 (55.3%)
Weight at baseline (kg)	74.9 (15.9)
Weight change (kg)	1.5 (5.6)

Table 2 shows the results of the linear marginal models, examining associations of two walkability indices with weight change. None of the walkability indices were significantly associated with weight change.

**Table 2:** Prospective relationships of neighbourhood walkability index and SSW with weight change (*N* = 910)

	Coefficient (95% CI)
Neighbourhood walkability	0.01 (−0.42, 0.44)
SSW	−0.19 (− 0.55, 0.16)

All models accounted for clustering at the CCD level and adjusted for age, gender, educational attainment, work status, marital status, having children in the household, annual household income, car ownership, neighbourhood SES, and baseline weight. All exposure measures were standardized

None of the associations with the six components of walkability reached statistical significance (Table 3); the strongest association was observed between street integration with weight gain: one standard deviation increment in street integration was associated with 0.31 kg less weight gain (95% CI = − 0.66, 0.05, *p* = 0.09).

**Table 3:** Prospective relationships of walkability components with weight change (*N* = 910)

	Coefficient (95% CI)
Net residential density	0.26 (− 0.14, 0.67)
Intersection density	−0.13 (− 0.59, 0.32)
Land use mix	0.12 (− 0.26, 0.49)
Net retail area ratio	0.03 (−0.41, 0.46)
Gross population density	0.11 (−0.28, 0.51)
Street integration	−0.31 (− 0.66, 0.05)†

† *p* ≤ 0.10

All models accounted for clustering at the CCD level and adjusted for age, gender, educational attainment, work status, marital status, having children in the household, annual household income, car ownership, neighbourhood SES, and baseline weight. All exposure measures were standardized

## Discussion

This study examined associations of two walkability indices and their individual components with weight change over four years among adults in Adelaide, Australia. Neither walkability indices nor their components were significantly associated with weight change. This is consistent with the afore-mentioned review in which the majority of studies found no relationships between neighbourhood walkability and weight-related measures [4].

A recent review found urban sprawl to be more consistently associated with adults’ weight status, compared with neighbourhood walkability [4]. These two constructs differ in terms of the scale at which measures are derived. Walkability is often calculated within a small local area, such as a 1 km buffer, which is approximately 3 km^2^ (in the case of a circular buffer). In contrast, urban sprawl is a city-scale measure that covers an area much larger than local neighbourhoods. For instance, studies on urban sprawl and weight status were mostly conducted in the U.S.A., and used “county sprawl index” [25]. County is an administrative unit with the median size of 1600 km^2^, which can encompass a whole city [26]. A potential reason that obesity is associated with urban sprawl but not with neighbourhood walkability is that the latter may not detect routine behaviours such as commuting and shopping that may influence weight change. Given that about 60% of physical activity has been reported to occur outside of a local area, which was defined as within 800 m from home [27], neighbourhood walkability may focus on an area too small to capture behaviours relevant to residents’ obesity. A recent longitudinal study conducted in Australia supports this argument: Sugiyama et al. found distance from city centre (but not neighbourhood walkability) to be associated with waist circumference increase over four years [28]. These authors argued that a larger scale environment needs to be considered since people’s time spent in cars for commuting and shopping, which is known to be related to obesity [29], is to a large extent dependent on where they live within a city [28]. The marginal association found for street integration in this study may also indicate the relevance of a larger scale environment to residents’ weight gain. A study using household travel survey data shows that street integration (measured at a local scale) is associated with car use (typically used to go beyond the local area), suggesting its capacity to predict behaviours that occur outside of the local area [30]. Space syntax measures, which are concerned with how a particular street is linked to other streets, appear to be inherently linked to macro characteristics of areas such as accessibility and remoteness. Space syntax has been indeed used in several studies to measure the level of sprawl [31, 32]. Further research is needed to develop a new environmental indicator that can better predict residents’ weight gain, building on existing potential measures such as sprawl, distance to city centre, and street integration.

This study has some limitations. Self-reported measure of weight may be subject to recall error and bias. Walkability indices and their components were calculated for each CCD, which had different sizes. Environmental measures, in particular intersection density, may be affected by varied CCD size, as previous studies have shown that intersection density tends to decrease as the area size increases [33, 34]. Further studies using individual buffers around each participant is required to confirm the results of this study. This study did not consider other locations, such as workplace, where people’s habitual physical activity may also influence weight gain over time. Future research could examine the impact of the attributes of workplaces and surrounding environments on weight gain. The study was conducted in Adelaide, thus the findings may be due to specific spatial characteristics of the city. In particular, as shown in an international study in which the same data from Adelaide were used [35], the city appears to have small variability in environmental attributes, which may have contributed to the non-significant associations observed. The study also did not consider food environment (access to healthy/unhealthy food). In addition, a relatively low response rate in the baseline and low retention rate may introduce some bias.

## Conclusions

In conclusion, this study suggests that research on obesity and the built environment may need to consider not just local environments but also a larger scale environment within a city or workplace environments in order to capture multiple behaviours relevant to weight gain. Research needs to develop such a new urban design measure to help identify areas where residents are more likely to have a greater risk of developing obesity.

## Abbreviations

CCD: Census Collection Districts
GIS: Geographic information systems
PLACE: The Physical Activity in Localities and Community Environments
SES: Socioeconomic status
SSW: Space syntax walkability

## References

[1] World Health Organization. Obesity and overweight. Fact sheet [updated June 2016]. http://www.who.int/mediacentre/factsheets/fs311/en/. Accessed 7 Dec 2017.

[2] Gortmaker SL, Swinburn BA, Levy D, Carter R, Mabry PL, Finegood DT, Huang T, Marsh T, Moodie ML (2011). Changing the future of obesity: science, policy, and action. Lancet.

[3] Sallis JF, Floyd MF, Rodríguez DA, Saelens BE (2012). Role of built environments in physical activity, obesity, and cardiovascular disease. Circulation.

[4] Mackenbach JD, Rutter H, Compernolle S, Glonti K, Oppert J-M, Charreire H, De Bourdeaudhuij I, Brug J, Nijpels G, Lakerveld J: Obesogenic environments: a systematic review of the association between the physical environment and adult weight status, the SPOTLIGHT project. BMC Public Health 2014, 14(1):233. 10.1186/1471-2458-14-233 PMC4015813 24602291

[5] Papas MA, Alberg AJ, Ewing R, Helzlsouer KJ, Gary TL, Klassen AC (2007). The built environment and obesity. Epidemiol Rev.

[6] Xu Y, Wen M, Wang F (2015). Multilevel built environment features and individual odds of overweight and obesity in Utah. Appl Geogr.

[7] Carlson JA, Saelens BE, Kerr J, Schipperijn J, Conway TL, Frank LD, Chapman JE, Glanz K, Cain KL, Sallis JF (2015). Association between neighborhood walkability and GPS-measured walking, bicycling and vehicle time in adolescents. Health & Place.

[8] Owen N, Cerin E, Leslie E, duToit L, Coffee N, Frank LD, Bauman AE, Hugo G, Saelens BE, Sallis JF (2007). Neighborhood walkability and the walking behavior of Australian adults. Am J Prev Med.

[9] Kaczynski AT, Glover TD (2012). Talking the talk, walking the walk: examining the effect of neighbourhood walkability and social connectedness on physical activity. Journal of Public Health.

[10] Van Dyck D, Cardon G, Deforche B, Sallis JF, Owen N, De Bourdeaudhuij I (2010). Neighborhood SES and walkability are related to physical activity behavior in Belgian adults. Prev Med.

[11] Feng J, Glass TA, Curriero FC, Stewart WF, Schwartz BS. The built environment and obesity: a systematic review of the epidemiologic evidence. Health & place. 2010;16 10.1016/j.healthplace.2009.09.008 19880341

[12] Sugiyama T, Koohsari MJ, Mavoa S, Owen N (2014). Activity-friendly built environment attributes and adult adiposity. Curr Obes Rep.

[13] Koohsari MJ, Owen N, Cerin E, Giles-Corti B, Sugiyama T (2016). Walkability and walking for transport: characterizing the built environment using space syntax. Int J Behav Nutr Phys Act.

[14] Australian Bureau of Statistics (2001). Census tables: Adelaide (statistical division).

[15] Harrison R, Cock D (2004). Increasing response to a postal survey of sedentary patients - a randomised controlled trial [ISRCTN45665423]. BMC Health Serv Res.

[16] Barnett AG, Van Der Pols JC, Dobson AJ (2004). Regression to the mean: what it is and how to deal with it. Int J Epidemiol.

[17] Ding D, Sugiyama T, Winkler E, Cerin E, Wijndaele K, Owen N (2012). Correlates of change in adults’ television viewing time: a four-year follow-up study. Sort.

[18] Leslie E, Coffee N, Frank L, Owen N, Bauman A, Hugo G (2007). Walkability of local communities: using geographic information systems to objectively assess relevant environmental attributes. Health & Place.

[19] Frank LD, Pivo G (1994). Impacts of mixed use and density on utilization of three modes of travel: single-occupant vehicle, transit, and walking. Transp Res Rec.

[20] Jiang B (2012). Axwoman 6.0: An ArcGIS extension for urban morphological analysis.

[21] Turner A: Depthmap 4, a researcher's handbook'. In*.*; 2004.

[22] Sugiyama T, Ding D, Owen N (2013). Commuting by car: weight gain among physically active adults. Am J Prev Med.

[23] Campmans-Kuijpers MJ, Sluijs I, Nöthlings U, Freisling H, Overvad K, Boeing H, Masala G, Panico S, Tumino R, Sieri S (2016). The association of substituting carbohydrates with total fat and different types of fatty acids with mortality and weight change among diabetes patients. Clin Nutr.

[24] Ball K, Crawford D, Ireland P, Hodge A (2003). Patterns and demographic predictors of 5-year weight change in a multi-ethnic cohort of men and women in Australia. Public Health Nutr.

[25] Berrigan D, Tatalovich Z, Pickle LW, Ewing R, Ballard-Barbash R (2014). Urban sprawl, obesity, and cancer mortality in the United States: cross-sectional analysis and methodological challenges. Int J Health Geogr.

[26] United States Census Bureau: USA counties data file. In: United States Census Bureau; 2010.

[27] Hillsdon M, Coombes E, Griew P, Jones A (2015). An assessment of the relevance of the home neighbourhood for understanding environmental influences on physical activity: how far from home do people roam?. Int J Behav Nutr Phys Act.

[28] Sugiyama T, Niyonsenga T, Howard NJ, Coffee NT, Paquet C, Taylor AW, Daniel M (2016). Residential proximity to urban centres, local-area walkability and change in waist circumference among Australian adults. Prev Med.

[29] Sugiyama T, Wijndaele K, Koohsari MJ, Tanamas SK, Dunstan DW, Owen N (2016). Adverse associations of car time with markers of cardio-metabolic risk. Prev Med.

[30] Koohsari MJ, Owen N, Cole R, Mavoa S, Oka K, Hanibuchi T, Sugiyama T (2017). Built environmental factors and adults' travel behaviors: role of street layout and local destinations. Prev Med.

[31] Volchenkov D, Blanchard P (2008). Scaling and universality in city space syntax: between Zipf and Matthew. Physica A: Statistical Mechanics and its Applications.

[32] Yang T, Hillier B: The fuzzy boundary: the spatial definition of urban areas. In: Proceedings, 6th International Space Syntax Symposium, İstanbul, 2007: 2007: Istanbul Technical University; 2007: 091001-091.016.

[33] Knight PL, Marshall WE (2015). The metrics of street network connectivity: their inconsistencies. Journal of Urbanism: International Research on Placemaking and Urban Sustainability.

[34] Stangl P. Overcoming flaws in permeability measures: modified route directness. Journal of Urbanism: International Research on Placemaking and Urban Sustainability. 2017:1–14.

[35] Adams MA, Frank LD, Schipperijn J, Smith G, Chapman J, Christiansen LB, Coffee N, Salvo D, du Toit L, Dygrýn J (2014). International variation in neighborhood walkability, transit, and recreation environments using geographic information systems: the IPEN adult study. Int J Health Geogr.

